# Acute Coronary Syndromes and Inflammatory Bowel Disease: The Gut–Heart Connection

**DOI:** 10.3390/jcm10204710

**Published:** 2021-10-14

**Authors:** Ayman Jaaouani, Abdulrahman Ismaiel, Stefan-Lucian Popa, Dan L. Dumitrascu

**Affiliations:** 1 Faculty of Medicine, “Iuliu Hatieganu” University of Medicine and Pharmacy, 400006 Cluj-Napoca, Romania; ayman.jaaouani.aj@gmail.com; 2 2nd Department of Internal Medicine, “Iuliu Hatieganu” University of Medicine and Pharmacy, 400006 Cluj-Napoca, Romania; popa.stefan@umfcluj.ro (S.-L.P.); ddumitrascu@umfcluj.ro (D.L.D.)

**Keywords:** acute coronary syndromes (ACS), inflammatory bowel disease (IBD), Crohn’s disease, ulcerative colitis, systematic review

## Abstract

(1) Background: Inflammatory bowel disease (IBD) induces a process of systemic inflammation, sharing common ground with acute coronary syndromes (ACS). Growing evidence points towards a possible association between IBD and an increased risk of ACS, yet the topic is still inconclusive. Therefore, we conducted a systematic review aiming to clarify these gaps in the evidence. (2) Methods: We conducted a systematic search on EMBASE, Cochrane Library, and PubMed, identifying observational studies published prior to November 2020. The diagnosis of IBD was confirmed via histopathology or codes. Full articles that fulfilled our criteria were included. Quality assessment was performed using the Newcastle–Ottawa scale (NOS). (3) Results: We included twenty observational studies with a total population of ~132 million subjects. Fifteen studies reported a significant association between ACS and IBD, while the remaining five studies reported no increase in ACS risk in IBD patients. (4) Conclusions: ACS risk in IBD patients is related to hospitalizations, acute active flares, periods of active disease, and complications, with a risk reduction during remission. Interestingly, a general increase in ACS risk was reported in younger IBD patients. The role of corticosteroids and oral contraceptive pills in increasing the ACS risk of IBD patients should be investigated.

## 1. Introduction

Inflammatory bowel disease (IBD), comprising Crohn’s disease (CD) and Ulcerative colitis (UC), outlines the incurable chronic inflammation of the gastrointestinal tract affecting approximately 2.2 million people in Europe [1] and 7.7 million Americans [2], whilst in Asia the incidence is 1.4 per 100,000 [3]. Nevertheless, the discrepancies in healthcare infrastructure and epidemiological reporting should be considered.

The number of patients with IBD is growing exponentially and is expected to significantly increase in the western world. Previously, IBD was thought to be limited to Caucasians in western countries but not anymore, as IBD was found to be rather related to environmental factors than ethnicity or heredity since most people do not present a family history and twin studies have not proven any concordance [4]. In addition, the incidence of IBD differs between regions in which the genetic background is similar. An increase in IBD incidence and prevalence in newly industrialized countries is being explained by the populations’ shift towards urbanism, lifestyle changes such as smoking and diet, and increased exposure to pollution and sedentarism [5].

CD and UC mostly affect young adults and adolescents between 20–30 and 30–40 years, respectively, although they have been reported to show a second peak at 60–70, showing a bi-modal distribution with the incidence of UC twice that of CD [5]. Smoking and appendectomy are risk factors demonstrated to affect the IBD risk [6]. Taking into consideration the demographics most affected by this condition and the relapsing course of this disease, chronic management is necessary. Although not yet fully understood, IBD pathogenesis involves a series of pathologic immune-mediated processes in individuals with a genetic predisposition [7].

Inflammation, being a key process in IBD, is also involved at all levels of coronary atherosclerosis and acute coronary syndromes (ACS), from the initial plaque formation to the thrombus rupture. It is worth noting the well-established association between IBD and the increased risk of venous thromboembolism due to the pro-atherogenic nature of the disease, especially during active flares [1].

ACS is the clinical manifestation of acute myocardial ischemia or infarction. ACS encompasses non-ST elevation ACS (NSTE-ACS), non-ST elevation myocardial infarction (NSTEMI), unstable angina (UA), and ST-elevation ACS (STE-ACS) [8].

Recently, several studies have cast light on the possible association between IBD and the increased risk of ACS. However, these assertions remain disputed as other studies claim no association. Therefore, we conducted the first systematic review to the best of our knowledge evaluating whether IBD patients are associated with an increased risk of ACS.

## 2. Materials and Methods

This systematic review was written following the preferred reporting items for systematic reviews and meta-analyses (PRISMA) guidelines [9].

### 2.1. Data Sources and Search Strategy

The electronic databases PubMed, EMBASE, and Cochrane Library were searched without any restrictions from their inception until November 4th 2020 to identify potential observational studies. The following search string was entered for PubMed ((“Acute Coronary Syndrome”[Mesh]) OR (“Acute Coronary Syndrome”[All Fields]) OR (“Myocardial Infarction”[Mesh]) OR (“Myocardial Infarction”[All Fields]) OR (“ST Elevation Myocardial Infarction”[Mesh]) OR (“ST Elevation Myocardial Infarction”[All Fields]) OR (“STEMI”) OR (“Non-ST Elevated Myocardial Infarction”[Mesh]) OR (“Non-ST Elevated Myocardial Infarction”[All Fields]) OR (“non-ST elevated myocardial infarction”) OR (“NSTEMI”)) AND ((“Inflammatory Bowel Diseases”[Mesh]) OR (“Inflammatory Bowel Diseases”[All Fields]) OR (“Crohn Disease”[Mesh]) OR (“Crohn Disease”[All Fields]) OR (“Colitis, Ulcerative”[Mesh]) OR (“Colitis, Ulcerative”[All Fields])), and similar search terms were used for EMBASE and Cochrane Library. Furthermore, we manually sorted the pertinent results across the three databases with the purpose of reducing results bias.

### 2.2. Study Selection and Eligibility Criteria

Observational studies assessing the association between ACS and IBD were eligible for inclusion. The original articles were included in the systematic review and qualitive assessment if they satisfied the following criteria: (1) Observational-study, population/hospital/primary care-based; (2) Inflammatory bowel disease confirmed by histopathology or ICD codes; (3) ACS diagnosis based on the criteria established in each study; (4) Studies on humans solely.

Exclusion criteria: (1) Experimental studies; (2) Studies published in languages other than English, German, or Romanian; (3) Case reports, letters, reviews, short surveys, practice guidelines, press articles, conference abstracts/papers; (4) Abstracts published without full-text or with the paper unavailable.

Two investigators (A.J. and A.I.) evaluated the titles and abstracts so that studies satisfying the inclusion and exclusion criteria were further assessed by reviewing the full paper, while in the case of discrepancies between the two investigators, a consensus was reached through discussion. Irrelevant studies were excluded.

### 2.3. Data Extraction

We extracted the following data from the included studies: author’s name, publication year, country, total subjects, study population, ACS patients, ACS mortality, IBD patients including CD and UC, mean age, gender distribution, IBD severity, treatment, follow-up duration, and the main findings. Data were extracted and entered by A.J while S.L.P. reviewed the extracted data for possible inaccuracies. Any discrepancies regarding the outcome of the data extraction were resolved through discussion. Extracted data were entered in a Spreadsheet while the final data were aggregated into the presented manuscript.

### 2.4. Quality Assessment

The investigators (A.J. and S.L.P.) independently used the Newcastle–Ottawa scale (NOS) in order to objectively assess the bias risk and internal validity of the included studies [10]. Separate assessment forms were used for case–control studies, cohort studies, and cross-sectional studies. Studies were scored based on how many stars were obtained and criteria were verified in the selection, comparability, and outcome section and thereafter graded accordingly following the evaluation, with scores ranging between 0 and 9 stars. Studies that received 7 stars or more were of high quality. The number of stars were added up in each study in order to compare the quality of included studies in a quantitative manner. Any discrepancies between the two investigators regarding the quality assessment of the included studies were discussed and a consensus was reached.

## 3. Results

### 3.1. Literature Search

**Figure 1:**
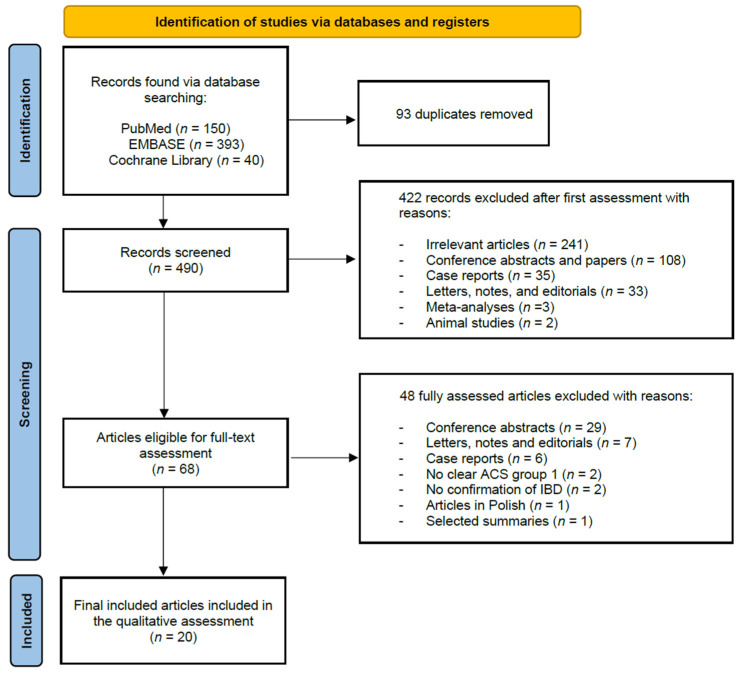
PRISMA flow diagram describing the identification, screening, and inclusion phases of our systematic review.

Figure 1 outlines the PRISMA flow diagram describing the identification, screening, and inclusion phases. The search strategy identified 583 articles throughout the three databases, of which 150 results were from PubMed, 393 from EMBASE, and 40 from Cochrane Library. A total of 93 duplicates were identified and removed. After a title and abstract review, we excluded 422 articles after first assessment. A total of 68 articles were retained for full-text evaluation according to the inclusion and exclusion criteria, of which 48 were excluded due to the following reasons: (1) No clear ACS group (*n* = 2) [11,12]; (2) Manuscripts in Polish (*n* = 1) [13]; (3) Letters, notes, and editorials (*n* = 7) [14,15,16,17,18,19,20]; (4) Case reports (*n* = 6) [21,22,23,24,25,26]; (5) No confirmation of IBD (*n* = 2) [27,28]; (6) Conference abstracts (*n* = 29) [29,30,31,32,33,34,35,36,37,38,39,40,41,42,43,44,45,46,47,48,49,50,51,52,53,54,55,56,57]; and (7) Selected summary (*n* = 1) [58]. In our final qualitative assessment, a total of 20 articles were included [1,2,4,7,59,60,61,62,63,64,65,66,67,68,69,70,71,72,73,74].

### 3.2. Study Characteristics

The summarized characteristics of the 20 included studies are shown in Table 1. About 132 million subjects are included in this study where IBD cases ranged from 80 to 563,687.

There were thirteen cohort studies (population-based cohort study [71], retrospective cohort study [1,2,61,62,63,64,65,66,72,73,75], cohort study [7]), four cross-sectional studies (cross-sectional study [59,60], retrospective cross-sectional study [67,69]), and three case–control studies [22,68,70].

Five studies were conducted in Europe (France *n* = 1, Greece *n* = 1, Denmark *n* = 2, UK *n* = 1), two studies in Asia (Taiwan *n* = 1, South Korea *n* = 1), and thirteen in North America (USA *n* = 13).

**Table 1:** Studies evaluating the risk of acute coronary syndromes in inflammatory bowel disease patients.

First Author/Year/ Country	Study Design	Study Characteristics	Main Findings
Mendelsohn et al./1995/USA [59]	Cross-sectional	• **Total Subjects:** 80 • **Population:** Crohn’s disease patients (deaths) • **ACS patients:** MI: 3 (12%) • **ACS mortality:** • **IBD:** Crohn’s disease: 25 (100%) • **Mean age (years):** - • **Gender (males):** 15 (60%) • **IBD severity:** • **Treatment:** operated (22 (88%) • **Follow up:** -	MI was associated with the death of three out of twenty-five patients with CD. Two patients who died from MI had hypertension possibly aggravated by multiple courses of steroid therapy.
Archimandritis et al./2002/Greece [60]	Cross-sectional	• **Total Subjects:** 172 • **Population:** IBD patients on follow-up • **ACS patients:** 4 (2.32%); UC: 2 (1.5%), CD: 2 (5%) • **ACS mortality:** 4 (100%) • **IBD:** 172 (100%); UC: 130 (67%); CD: 42 (42%) • **Mean age (years):** UC (men: 46.00 ± 16.24, women: 40.00 ± 16.76)/CD (men: 36.85 ± 13.0, women: 33.79 ± 16.9)) • **Gender (males):** 95 (55.8%); UC: 73 (56.1%); CD: 22 (52.3%) • **IBD severity:** - • **Treatment:** UC (conservative: 108 responded well vs. 22 did not respond well; surgical: 6); CD (known drug regimen and individualized: 42, required surgery: 12) • **Follow up:** UC: 25% of patients experienced a severe attack and 17% had their bowel involvement worsen	The mortality rate for UC was 5%, but only 1.5% could be directly connected with the disease; the rate was 5% in CD, unrelated to the disease. The only two men who died while having CD had an MI.
Ha et al./2009/USA [61]	Retrospective cohort analysis	• **Total Subjects:** 17,487 • **Population:** IBD patients (code: ICD-9CM) aged between 18–59 years, from the MarketScan Commercial claims and Encounters database • **ACS patients:** IBD: 148 (0.9%); UC: 83 (0.8%); CD: 65 (0.9%) • **ACS mortality:** - • **IBD:** 17,487 (100%); UC: 9968 (57%), CD: 7480 (42,77%) • **Mean age (years):** UC: 43.6, CD: 42.9; Control: 43.2 (18–59)44 • **Gender (males):** UC: 4455 (44.7%); CD: 3254 (43.5) • **IBD severity:** - • **Treatment:** - • **Follow up:** UC: 3.2 (0.5–5.7) years; CD: 3.3 (0.5–5.7) years	Only IBD women between 40–59 showed an elevated risk for MI, while for men over 40 there was no increase and a significant lower risk of atherosclerosis. A higher percentage of women used contraceptives vs. age-adjusted control group.
Pemmasani et al./2020/USA [73]	Retrospective cohort analysis	• **Total Subjects:** • **Population:** ACS patients • **ACS patients:** ACS: 6,896,635 (100%) • **ACS mortality:** IBD related comorbidities and complications associated with increased mortality • **IBD:** 24,200 (0.35%); CD: 12,846 (53%); UC: 11,374 (47%) • **Mean age (years):** No IBD: 67.2 ± 14.4; IBD: 66.9 ± 13.4 • **Gender (males):** 4,130,321 (60.1%) • **IBD severity:** - • **Treatment:** - • **Follow up:** -	ACS-related risk profiles and mortality were more favorable with IBD-ACS than with non-IBD ACS. Comorbidities and complications more frequently associated with IBD were strongly associated with mortality from ACS. Among IBD patients with ACS, comorbidities and complications that were potentially related to IBD were strong independent predictors of increased mortality.
Osterman et al./2011/USA [62]	Retrospective cohort analysis	• **Total Subjects:** 25,327 • **Population:** Patients with CD or UC older than 18 years old, no history of RA, SLE, psoriasis, MI, CAD, CHF, ventricular arrhythmia, cardiac defibrillator implantation before the start of the follow-up, pulled from the General Practice Research Database • **ACS patients:** IBD: 390 ; UC: 280 (1.8%) ; CD: 110 (1.1%) • **ACS mortality:** - • **IBD:** 100%, UC: 61.2% (15,498); CD: 38.8% (9829) • **Mean age (years):** UC: 50 vs. 49.1 from general population; CD: 44.2 vs. 43.3 from general population • **Gender (males):** UC: 48.4% (7501); CD: 41% (4030) • **IBD severity:** - • **Treatment:** - • **Follow up:** UC f/u: mean of 4.6 years; CD f/u: mean of 4.4 years	Patients with UC or CD do not appear to be at increased risk of MI. These results are contrary to those seen in other chronic inflammatory diseases, such as RA, SLE, and psoriasis.
Merril et al./2012/USA [63]	Retrospective cohort study	• **Total Subjects:** 271,368 • **Population:** Patients with inflammatory bowel disease (IBD) undergoing surgery • **ACS patients:** 9 (0.4%) PS: MI and CVA, study does not provide separate numbers • **ACS mortality:** - • **IBD:** 2249 (0.8%) • **Mean age (years):** IBD: 43; Non-IBD: 55.5 • **Gender (males):** 1122 (49.9%) • **IBD severity:** - • **Treatment:** - • **Follow up:** -	This analysis revealed no association between IBD and perioperative MI and stroke.
Kristensen et al./2013/Denmark [64]	Retrospective cohort study	• **Total Subjects:** 20,795 • **Population:** IBD cases ≥ 15 years old who received first diagnosis of IBD during 1996–2009 with dispensed IBD treatment prescription with no prior IBD or MI or stroke before that period • **ACS patients:** 365; UC: 272 (74.5%); CD: 61 (16.7%) *unspecified IBD = 32* (*8*%) • **ACS mortality:** IBD: 778 (3.74%); UC: 540 (69.4%); CD: 148 (19%) 11% is unspecified IBD. Mentions cardiovascular death in IBD patients not ACS or MI explicitly • **IBD:** 100% UC: 13,622 (65.5%); CD: 4732 (22.8%) • **Mean age (years):** IBD: 43.8 years (SD: 18.7); Control: 43.1 (18.7SD) • **Gender (males):** 45.5% (9462) • **IBD severity:** - • **Treatment:** Anti-TNF and corticosteroids • **Follow up:** Mean f/u time is 6.04 years	IBD patients were found to have a significantly increased (two-fold) risk of MI, stroke, and cardiovascular mortality. This risk was predominantly present in periods of IBD flares and persistent activity, whereas the risk was insignificantly raised for MI and stroke and not increased for cardiovascular death during remission disease stages.
Aggarwal et al.//2014/USA [65]	Retrospective cohort study	• **Total Subjects:** 131 • **Population:** Patients with IBD who were diagnosed with CAD by cardiac catheterization between January 2004 and June 2010 • **ACS patients:** 31 (23.66%) • **ACS mortality** *has a death parameter but associated with CAD not ACS specifically*. • **IBD:** 131 (100%); UC: 77 (58.77%); CD: 54 (41.22%) • **Mean age (years):** 65.3 years (10.0SD); UC: 64.8 (10.8SD); CD: 66.1 (8.7 SD); non-IBD: 67.8 (11.0SD) years • **Gender (males):** 97 (74.04%); UC: 34 (63%); CD: 63 (81.8%) • **IBD severity:** - • **Treatment:** Amino-salicylates, immunomodulators (any use of 6-mercaptopurine, azathioprine, or methotrexate), corticosteroids (oral or intravenous steroidal agents), topical therapies (enemas or suppositories), or biologics (any use of infliximab, adalimumab, or certolizumab) • **Follow up:** Median follow-up was 12 months (post-PCI)	Patients with IBD are diagnosed with CAD at a younger age as compared with non-IBD patients, are less likely to be active smokers and have lower body mass index. There was no difference in post-PCI major adverse cardiovascular outcomes.
Kristensen et al./2014/Denmark [1]	Retrospective cohort study	• **Total Subjects:** 73,451 • **Population:** Patients aged ≥ 30 years old hospitalized for the first-time MI between 2002–2011 alive 30 days post-discharge • **ACS patients:** 100%. • **ACS mortality:** 270 *all-cause death* • **IBD:** 863 (1,17%); UC: 655 (75.9%); CD: 208 (24.1%) • **Mean age (years):** IBD: 68.5 (13.5SD); No-IBD: 68.4 (13.7) • **Gender (males):** 498 (57.7%) • **IBD severity:** - • **Treatment:** Corticosteroid, anti-TNF • **Follow up:** Mean follow-up for patients with IBD alive 30 days after their first-time MI is: 3.9 years	Patients with IBD have increased long-term risk of all-cause mortality and major adverse cardiovascular events after MI, and this risk is exclusively observed during active IBD, in particular in relation with flare-ups.
Tsai et al./2014/Taiwan [66]	Retrospective cohort study	• **Total Subjects:** 11,822 • **Population:** Patients with IBD symptoms • **ACS patients:** 434; UC: 162 (37.32%); CD: 272 (62.68%) • **ACS mortality:** - • **IBD:** 100% *does not specify numbers by UC and CD* • **Mean age (years):** control: 52.3 (19.2SD); IBD:52.8 (19.3SD) • **Gender (males):** 6428 (54.4%) • **IBD severity:** - • **Treatment:** - • **Follow up:** Mean follow-up periods = 6.37 (3.76SD) years	The patients with IBD in this study were more likely to exhibit traditional risk factors for ACS. Patients with IBD are at elevated risks of deaths from myocardial infarction, stroke, and cardiovascular disorders. Patients with IBD who, on average, required two or more hospitalization per year were nearly 20-fold more likely to have ACS than those who required one hospitalization per year.
Kuy et al./2014/USA [67]	Retrospective cross-sectional analysis	• **Total Subjects:** 461,415 • **Population:** Patients with IBD from 2000 to 2009 • **ACS patients:** 9197 (1.99%) • **ACS mortality:** - • **IBD:** 100% • **Mean age (years):** - • **Gender (males):** • **IBD severity:** - • **Treatment:** - • **Follow up:**	ATEs represent most clinically relevant thromboembolic complications associated with inpatient admissions of IBD patients.
Zakroysky et al./2015/USA [68]	Case-control study	• **Total Subjects:** 177 • **Population:** IBD patients with a first presentation of ACS • **ACS patients:** 59 (33.33%); STEMI (ST-ACS) = 25 (42%); NSTEMI (NST-ACS) = 25 (42%); Unstable-angina = 9 (15%) • **ACS mortality:** Two deaths from cardiac causes; Patients with inflammatory bowel disease with acute coronary syndrome had a significantly higher all-cause mortality than those without acute coronary syndrome (17% vs. 5%, OR 3.7; 95% CI, 1.3–11.0; P = 0.02) • **IBD:** 100%; UC: 99 (55.9%); CD: 78 (44.06%) • **Mean age (years):** ACS: 67 ± 10; No-ACS: 67 ± 10 • **Gender (males):** 132 (74.5%); ACS: 44 (75%); No-ACS: 88 (75%) • **IBD severity:** - • **Treatment:** Anti-inflammatory (Steroids); biological agent (Infliximab); immunomodifiers (azathioprine, 6-mercaptopurines, methotrexate) • **Follow up:** -	There is an association between steroid exposure and significantly reduced odds of acute coronary syndrome. However, the use of amino salicylates, immune modifiers, and biologic therapies did not affect acute coronary syndrome events.
Barnes et al./2016/USA [69]	Retrospective cross-sectional study	• **Total Subjects:** - • **Population:** Patients ≥ 18 years diagnosed with IBD between 2000–2011 • **ACS patients:** - • **ACS mortality:** - • **IBD:** 563,687 (0.71%); UC: 204,589 (36.3%); CD: 359,098 (63.7%) • **Gender (males):** IBD: 237,111 (42.1%); Without-IBD: 30,623,519 (39.2%) • **IBD severity:** - • **Treatment:** - • **Follow up:** -	Patients with IBD had 0.51-fold odds of diagnosis of acute MI compared with patients without IBD. Patients with UC were more likely to have a diagnosis of acute MI than patients with CD. Lower rates of acute MI were demonstrated in the IBD population when compared with the general population (nationwide database).
Ehrenpreis et al./2016/USA [70]	Case-control	• **Total Subjects:** 5349 • **Population:** patients with ICD-9 cm codes for primary diagnosis of acute myocardial infarction, pneumonia or congestive heart failure with a co-diagnosis of IBD, Crohn’s disease (CD) or ulcerative colitis (UC). 2005–2011 NIS Database • **ACS patients:** 2280 (42.62%); CD: 1164 (51.05%); UC: 1123 (48.95%) • **ACS mortality:** 94; CD: 47 (50%); UC: 47 (50%) • **IBD:** UC: 1985 (37.10%); CD: 3364 (62.89%) • **Mean age (years):** UC: 65.79 ± 17.96; CD: 61.32 ± 17.88 • **Gender (males):** IBD: 2328 (43.5%) UC: 997 (50.23% out of UC patients); CD: 1331 (39.57% out of CD patients) • **IBD severity:** - • **Treatment:** - • **Follow up:** -	IBD confers a survival benefit for patients hospitalized with AMI.
Aniwan et al./2018/USA [71]	Population-based cohort study	• **Total Subjects:** 736 • **Population:** Patients with IBD in Olmsted County, Minnesota from 1980 through 2010 • **ACS patients:** 75 (10.19%) • **ACS mortality:** - • **IBD:** 100%; CD: 339 (46.05%); UC: 397 (53.94%) • **Mean age (years):** - • **Gender (males):** IBD: 405 (55%); CD: 177 (52%); UC: 228 (57%) • **IBD severity:** Systemic corticosteroids and IBD-related intraabdominal surgery as markers of disease severity and not scores • **Treatment:** Systemic corticosteroids, biologics, intraabdominal surgery • **Follow up:** -	The relative risk of AMI was significantly increased in patients with Crohn’s disease and ulcerative colitis. The relative risk of AMI was increased among users of systemic corticosteroids. Patients with IBD are at increased risk of AMI and heart failure.
Le Gall et al./2018/France [22]	Case-control study	• **Total Subjects:** 3539 • **Population:** All patients, aged 18 or older with an occurrence of acute arterial event between 1996 and 2015 the MICISTA database. Only patients with a follow-up greater than one year and at least one visit per year in our IBD unit. • **ACS patients:** 22 (0.63%) • **ACS mortality:** - • **IBD:** 100% • **Mean age (years):** [nested case–control] median Cases: 41.9 (25.3–58.7) vs. Control: 43.3 (31.7–54.6) • **Gender (males):** [nested case–control] 18 (60% of 30 cases) • **IBD severity:** - • **Treatment:** Corticosteroids, 5 amino-salicylates, thiopurines, methotrexate, and anti–tumor necrosis factor agents [anti-TNFs] • **Follow up:** Follow-up greater than one year and at least one visit per year in our IBD unit	The median interval between IBD diagnosis and occurrence of acute arterial event was 15.4 years. Disease activity may have an independent impact on the risk of acute arterial events in patients with IBD.
Choi et al./2019/South Korea [7]	Cohort study	• **Total Subjects:** 37,477 • **Population:** Patients diagnosed with Crohn’s disease (CD) or ulcerative colitis (UC) between 2006 and 2009 • **ACS patients:** Total: 604 (1.6%); CD: 146 (1.36); UC: 440 (1.64) • **ACS mortality:** *the table mentions death but not specifically mentions if the death was due to ACS* • **IBD:** 100%; CD: 10,708 (28.57%); UC: 26,769 (71.43%) • **Mean age (years):** IBD: 40.4 ± 16.6; Control: 40.4 ± 16.6; CD: 32.5 ± 15.7; UC: 43.5 ± 15.9 • **Gender (males):** 21,293 (56.8%); CD: 6881 (64.3%); UC: 14,412 (53.8%) • **IBD severity:** - • **Treatment:** Surgery (related to IBD: bowel resection) • **Follow up:** Median follow-up durations of control and IBD groups were 8.4 ± 1.6 years	The risk of MI is higher in patients with CD than in the general population, and this trend is stronger in female patients and those aged <40 years.
Panhwar et al./2019/USA [2]	Retrospective cohort analysis	• **Total Subjects:** 29,090,220 • **Population:** adult patients (20 to 65 years) with a diagnosis of IBD—ulcerative colitis (UC) or Crohn’s disease (CD)—who had active records between August 2013 and August 2018 • **ACS patients:** IBD: 20,040 (6.9%) UC: 9086 (45.33%) vs. CD 10,954 (54.66%) • **ACS mortality:** - • **IBD:** 290,430 (0.99%); UC: 131,680 (0.45%); CD: 158,750 (0.55%) • **Mean age (years):** (*UC or CD were less likely to be younger* (*20–65 years old*) • **Gender (males):** IBD: 11,6967 (40.27%); UC: 53,549 (40.7%); CD: 63,373 (39.92%) • **IBD severity:** - • **Treatment:** - • **Follow up:** -	IBD is associated with significantly increased MI risk compared with non-IBD patients. The relative risk of MI was highest in younger patients and decreased with age. The prevalence of MI was higher in patients with UC and CD vs. non-IBD patients. Patients with CD had greater odds of MI compared with patients with UC (across all age groups). Male gender conferred higher risk of MI.
Card et al./2020/UK [72]	Retrospective cohort study	• **Total Subjects:** 31,175 • **Population:** IBD patients with no restriction on age, no history of CAD, TEE or malignancy before diagnosis and no other steroids indication from the CPRD database, between 1997 and 2017 • **ACS patients:** 532 (1.7%) • **ACS mortality:** *mentions 469 cardiovascular deaths, not exactly as a result from MIs/ACS* • **IBD:** 31,175 (100%); UC: 16,779 (53.82%); CD: 10,721 (34.38%); Indeterminate-IBD: 3538 (11.34%) • **Mean age (years):** IBD: 45.2 vs. 45.4 Controls; UC: 47.8; CD: 41.5 • **Gender (males):** IBD: 14,883 (47.8%) vs. Control: 73,863 (47.8%); UC: 8359 (50%); CD: 4887 (44.8%); • **IBD severity:** - • **Treatment:** - • **Follow up:** Follow-up time according to disease activity; 220,000 person years vs. 1,000,000 person years in controls. MIs had 1.85 per 1000 person years when hospitalized	Hospitalized IBD patients had a lower risk of vascular events than controls, being significant only for MIs. An increased hazard of MI in ambulatory patients when disease was active. The incidence ratios for MIs were significantly increased in acute and chronic activity of IBD within ambulatory but not hospitalized patients.
Gauravpal S. Gill et al./2020/USA [75]	Retrospective Cohort study	• **Total Subjects:** 3,917,894 • **Population:** Patients with IBD from MedStar Health electronic record system pool of patients • **ACS patients:** No-IBD: 1.8% (277); IBD: 2.0% (302) • **ACS mortality:** Non-IBD: 2.1% (324) ; IBD: 2.3% (352) ; UC: 2.6% (171) ; CD: 2.2% (207) • **IBD:** 0.39% (15,292), UC: 43% (6658), CD: 61% (9406) • **Mean age (years):** IBD: 50; Non-IBD: 51; UC: 53; CD: 49 • **Gender (males):** Non-IBD: 41% (6337) ; IBD: 42% (6377) ; UC: 43% (2868); CD: 41% (3835) • **IBD severity:** - • **Treatment:** - • **Follow up:** Median follow-up of 4.4 years	Among patients with IBD, incidence of acute coronary events did not show a statistically significant difference when compared to the matched cohort.

MI: myocardial infarction; CD: Crohn’s disease; IBD: inflammatory bowel disease; UC: ulcerative colitis; ACS: acute coronary syndrome; ICD: international classification of diseases; RA: rheumatoid arthritis, SLE: systemic lupus erythematosus; CAD: coronary artery disease; CHF: congestive heart failure; CVA: cerebrovascular accident; TNF: tumor necrosis factor; SD: standard deviation; PCI: percutaneous coronary intervention, ATE: arterial thromboembolism; STEMI: ST elevation myocardial infarction; NSTEMI: non-ST elevation myocardial infarction; AMI: acute myocardial infarction; TEE: transesophageal echocardiogram.

### 3.3. Quality Assessment

We used the NOS for the quality assessment tool to assess the included studies in our review as illustrated in Supplementary Tables S1–S3. Eighteen studies had an overall rating of >7 stars [1,7,22,59,60,61,62,63,64,65,66,68,69,70,71,72,73,75] and three studies received 5–6 stars [2,65,67]. Several included studies did not report some criteria in the NOS quality assessment tool. Among the cross-sectional studies, two studies did not report the sample size [59,60], one study did not fulfill the comparability criterion, and two did not report statistical tests [60,67]. Regarding case–control studies, one study did not define controls [68]. Out of the cohort studies, eight studies presented the outcome of interest at the start [1,2,61,62,63,65,66,73], four studies did not report a long enough follow up [1,63,65,73], whereas follow-up was not possible in one study [2] and not adequate in another [65].

Twelve studies rated with a score > 7 stars reported a significant association between IBD and ACS [1,7,22,59,60,61,64,66,68,69,71,72], in contrast to five studies with the same rating that found the contrary [62,63,70,73,75]. All the studies that received 5–6 stars on evaluation showed a significant association between ACS and IBD [2,65,67].

### 3.4. Definition of IBD

Most studies confirmed the diagnosis of IBD using colonoscopy and histopathological examination or the International Classification of Disease (ICD) codes.

### 3.5. IBD Potentially Increasing the Odds of ACS Occurrence

Several studies evaluated whether IBD increases the risk of a patient developing ACS, with inconsistent results.

Mendelsohn et al. assessed 80 Crohn’s patients where 12% of the deaths were associated with myocardial infarction (MI) while suggesting that it might have been aggravated by steroid therapy [59]. A retrospective cohort analysis by Ha et al. evaluated 17,487 patients with IBD, concluding that women between 40–59 showed an increased risk of MI in comparison to their age matched groups [61]. Moreover, Kristensen et al. conducted two retrospective cohort studies, the first study involving patients > 15 years old with IBD and dispensed treatment. The authors reported that IBD patients were at a higher risk (two-fold) of MI, stroke, and cardiovascular mortality during episodes of flares and active disease but not during remission phases. The second study by Kristensen et al. involved 73,451 patients ≥ 30 years of age who were hospitalized for the first time for MI. The result was that patients with IBD, especially during flares, were shown to have a high risk of mortality from major adverse cardiovascular events after an MI [1,64]. Furthermore, Aggarwal et al. evaluated 131 patients with IBD, diagnosed with CAD via catheterization, demonstrating that IBD patients were diagnosed with CAD at a younger age while there was no major difference in post-PCI adverse outcomes [65]. A retrospective cohort study from Taiwan by Tsai et al. studied 11,822 patients with IBD symptoms [66] and found that IBD patients were more likely to present the traditional risk factors for ACS and that patients requiring two or more hospitalizations were almost 20-fold more likely to endure an ACS event than those requiring one hospitalization per year, and that IBD women were more likely to have an ACS in the adjusted HR, which further supports the results found by Kristensen et al. [64]. A retrospective cross-sectional study by Kuy et al. studied 461,415 IBD patients and reported an alarming increase in embolic events in these patients [67]. Barnes et al. conducted a nation-wide retrospective cross-sectional study with 78,684,687 subjects, demonstrating that IBD patients were younger and have 0.51-fold odds of having an MI compared to non-IBD subjects, while it was more likely to occur in UC compared to CD [69]. A population-based cohort study by Aniwan et al. involving 736 IBD patients in Olmsted County, Minnesota, reported an ACS percentage of 10.19% and a significant increase in relative risk of acute MI in these patients, with an even amplified risk with systemic corticosteroids [71]. The Le Gall et al. case–control study conducted on 3539 IBD patients suggested that the disease activity is an independent factor in the risk of an acute arterial event [22]. A South Korean cohort study by Choi et al. with 37,477 IBD patients demonstrated a higher risk of MI in CD, with a stronger trend in females [7]. Panhwar et al. conducted a retrospective cohort analysis with 29,090,220 participants, which concluded with a significantly higher risk of MI in younger IBD patients compared to non-IBD patients [2]. A retrospective cohort conducted by Card et al. studied 31,175 patients, reporting an increased hazard of MI in ambulatory active IBD and a lower hazard of MI in hospitalized patients [72].

On the other hand, Pemmasani et al. reported in their retrospective cohort study involving 6,896,635 ACS patients that patients with concurrent IBD had a modestly favorable CVD risk factor profile. However, the mortality from ACS was strongly associated with IBD complications, which are potentially considered strong independent predictors of increased mortality [73]. Moreover, Osterman et al. conducted a retrospective cohort analysis demonstrating that UC and CD patients were not at an increased risk of MI, unlike other chronic inflammatory diseases (RA, SLE, psoriasis, etc.) [62]. The cohort study performed by Merril et al. comprising 271,368 patients with IBD undergoing surgery concluded that there was no association between IBD and perioperative MI and stroke [63]. A case–control study conducted by Ehrenpreis et al. included 5349 patients with code for acute MI, pneumonia, and congestive heart failure with a co-diagnosis of IBD, reported that IBD confers survival benefits for patients hospitalized with acute MI and 34% survival in patients hospitalized for AMI with a CD co-diagnosis [70]. The retrospective cohort study with 3,917,894 patients where IBD percentage was 0.39% and ACS percentage among IBD patients was 2% conducted by Gauravpal et al. [75] demonstrated a statistically non-significant difference in the incidence between IBD and non-IBD patients, with the cardiovascular mortality being 2.3% and 2.1%, respectively.

## 4. Discussion

The issue of whether IBD increases the risk of ACS has been addressed in several studies with conflicting results. Our systematic review addresses this issue, as it includes twenty articles with a total study population of approximately 132 million individuals, out of which, fifteen studies reported an increased risk of ACS in IBD patients. All but two of these fifteen studies were rated with a score of >7 stars on quality assessment using NOS, while the remaining two received 5–6 stars on the quality assessment [65,67]. All five studies reporting no significant increase in ACS risk in IBD patients received >7 stars. Furthermore, the studies that reported a significant association between ACS and IBD nine of them were cohort studies, three cross-sectional and one case–control. On the other hand, among the studies that reported no significant association four were cohort studies and one case–control.

In our systematic review, several findings need to be elaborated. Several studies associated an increased risk of MI and morality in ambulatory IBD patients, coupled with other studies that claim a lower percentage of ACS in hospitalized IBD patients. In addition, the study by Tsai et al. reported that patients who needed two or more hospitalizations on average were 20-fold more likely to have ACS than those who required one hospitalization per year. Similarly, Barnes et al. found that IBD patients had 0.51-fold odds of having an acute MI as opposed to non-IBD patients. Furthermore, comorbidities and complications were reported to be independent predictors linked with increased risk and mortality from ACS in acute flares and fulminant stages with IBD. On the other hand, the risk of MI and cardiovascular events did not increase during the remission stages of the disease [1,22,64,72].

Studies that reported an increased percentage of ACS in young IBD patients had a younger study population in contrast to studies that reported no increased risk of ACS in IBD patients. The study conducted by Ha et al. showed that a high percentage of women that used oral contraceptive pills (OCP) had an elevated risk of MI. It is possible that there is a compounding of the risk brought by contraceptives, especially estrogen containing oral contraceptives [74], with the pro-inflammatory state of IBD. This could possibly explain the increased risk of ACS in young women with IBD.

In fact, the increased risk of ACS and mortality in active disease is consistent due to the role of systemic inflammation in increasing atherosclerosis being consistent with the high lipidic profile in IBD patients. Patients with IBD have an increased production of reactive oxygen species (ROS), increased expression of inflammatory cytokines (TNF-⍺ and IL-6) and antibodies that lead to vascular smooth muscle cell proliferation (VSMC), endothelial dysfunction, and the development of CVD [76]. This was illustrated in a recent study conducted by Hernández-Camba et al. that reported a higher frequency of IBD patients being reclassified into a very-high cardiovascular risk via ultrasound assessment of carotid plaques [77]. Interestingly, IBD patients see lower frequencies of acute MI when compared to other chronic inflammatory diseases such as rheumatoid arthritis and systemic lupus erythematosus, which might perhaps highlight a different pathophysiological mechanism where platelet dysfunction is more involved [69].

Two studies cited steroid therapy in IBD as an explanation for the increased relative risk of ACS [68,71]. However, steroids are used in the management of acute flares of IBD, and as stated earlier, several studies have demonstrated an increased risk of ACS in acute flares and the fulminant and active stages of IBD. It is possible that steroid use is not associated with the increased odds of ACS, but the severity of the disease in which they happen to be used more in, which is acute flares and severe IBD. Although the association of corticosteroid usage in IBD patients and ACS is controversial, it is hypothesized that steroids usage increases CVD risk through several pathways including the sympathetic stimulation of the renin–aldosterone–angiotensin axis [76]. All things considered, further studies should evaluate the benefit of immunosuppressive therapy and further investigate the association between corticosteroids and ACS in IBD patients and more aggressive anti-inflammatory therapies should be explored to reduce atherosclerosis, cardiovascular comorbidities, and mortality.

Furthermore, we noticed that two studies reached opposite findings. While Tsai et al.’s findings point towards an increased risk of ACS upon hospitalization, Card et al. claim that there is no significant increase. This discrepancy may have several explanations, one of which is that Card et al. excluded patients previously diagnosed with vascular disease while Tsai et al. only excluded patients hospitalized with a previous diagnosis of ACS. Another explanation could be the lack of data in Tsai et al.’s study that could represent a significant confounder such as the smoking history, family history of CAD, alcohol consumption, etc. Furthermore, Card et al. states that a possible explanation for the ~25% decreased risk of MI in IBD patients (attributed to hospitalization) in comparison to the controls might be since for many IBD patients hospitalization will be due to IBD and hence the admission related to vascular factors will represent a smaller proportion. In addition, Tsai et al. uses hospitalization as a surrogate marker for IBD severity, which could have created a bias towards sicker patients. Meanwhile, Card et al. uses corticosteroid prescription as a surrogate marker for IBD severity [66,72]. It is worth noting that six papers of the 20 included in this study discussed ACS mortality in IBD [1,60,68,70,73,75], but none assessed IBD severity using the CDAI for Crohn’s disease and DAI score for ulcerative colitis.

Our systematic review has several limitations. Although none of the included studies were of poor quality and only three studies were of fair quality [2,65,67], these ratings should be considered cautiously due to possible methodological bias and flaws. Due to the observational design of the studies included in our systematic review, causality between IBD and increased ACS risk cannot be confirmed. Most studies were of retrospective design, without having a follow-up period. Moreover, none of the studies assessed the severity of IBD, which did not allow us to assess the association between IBD staging and the risk of ACS. In our systematic review, we did not include studies published in the grey literature as they might lead to biases in our conclusions due to possible methodological flaws.

Regardless, our systematic review presents several strengths, including a large study population. The topic of our systematic review is of clinical relevance as the prevalence and incidence of IBD is exponential while the rates of newly industrialized nations are growing in parallel and the global burden of IBD on already limited healthcare systems with substantial therapies have significantly changed over the years, whilst more studies are shedding light on a possible increased risk of ACS in IBD patients. Furthermore, we conducted a comprehensive search, including the current studies evaluating our topic and summarizing the currently available data in a nonbiased manner while pointing out the missing data that need to be further assessed in future studies.

## 5. Conclusions

In conclusion, the risk of ACS increases significantly with hospitalization and acute active flares, in addition to prolonged periods of active disease. On the other hand, IBD patients in remission present with a lower risk for ACS. The general increased risk of ACS in young IBD patients, possibly due to corticosteroid use, in addition to the effects of estrogen containing OCPs in young IBD female patients, should be further investigated. The interplay between several risk factors including chronic inflammation, thrombosis, corticosteroid use, lipid and endothelial dysfunction, and gut dysbiosis are likely to play a crucial role in the association between IBD and increased ACS risk. A better understanding of these mechanisms may possibly lead to developing novel therapeutic targets in patients with IBD.

Managing IBD patients with ACS risk should be performed through a multidisciplinary team-based approach, while aiming to induce disease remission. Screening and management of cardiovascular risk factors are required, especially in IBD patients with increased risk. Future research is required to better elucidate the pathophysiological mechanisms behind the increased ACS risk in IBD patients. Moreover, further studies assessing the severity of IBD, aside from hospitalizations or corticosteroid prescriptions as surrogate markers for severity, in addition to the effect of biological agents in hospitalized IBD patients, on the risk of ACS remain necessary.

## Supplementary Materials

The following are available online at https://www.mdpi.com/article/10.3390/jcm10204710/s1, Table S1: The Newcastle-Ottawa Scale (NOS) for assessing the quality of cohort studies, Table S2: The Newcastle-Ottawa Scale (NOS) for assessing the quality of cross-sectional studies, Table S3: The Newcastle-Ottawa Scale (NOS) for assessing the quality of case-control studies.
