# Supplementary material for: Acute Coronary Syndromes and Inflammatory Bowel Disease: The Gut–Heart Connection
Source: J Clin Med. 2021 Oct 14;10(20):4710. doi: 10.3390/jcm10204710 (PMC8538087; doi:10.3390/jcm10204710)
Supplement: Supplementary file 1 [file jcm-10-04710-s001.zip › jcm-1413540 supplementary.pdf]

Table S1. The Newcastle-Ottawa Scale (NOS) for assessing the quality of cohort studies

Study	Selection				Comparability	Outcome			Score
	**Representativeness of the exposed (⋆)**	**Selection of non-Cohort (⋆)**	**Ascertainment of exposure (⋆)**	**Outcome of interest not presented at the start (⋆)**	**(⋆⋆)**	**Assessment of outcome (⋆)**	**Was the follow-up long enough (⋆)**	**Adequacy of the follow-up (⋆)**	
Ha et al. / 2009 / USA [61]	⋆	⋆	⋆	-	⋆⋆	⋆	⋆	⋆	(8)
Pemmasani et al. / 2020 / USA [73]	⋆	⋆	⋆	-	⋆⋆	⋆	-	*	(7)
Osterman et al. / 2011 / USA [62]	⋆	⋆	⋆	-	⋆⋆	⋆	⋆	⋆	(8)
Merril et al. / 2012 / USA [63]	⋆	⋆	⋆	-	⋆⋆	⋆	-	⋆	(7)
Kristensen et al. / 2013 / Denmark [64]	⋆	⋆	⋆	⋆	⋆⋆	⋆	⋆	⋆	(9)
Aggarwal et al. / / 2014 [65]	-	⋆	⋆	-	⋆⋆	⋆	-	-	(5)
Kristensen et al. / 2014 / Denmark [1]	⋆	⋆	⋆	-	⋆⋆	⋆	-	⋆	(7)
Tsai et al. / 2014 / Taiwan [66]	⋆	⋆	⋆	-	⋆⋆	⋆	⋆	⋆	(8)
Aniwan et al. / 2018 / USA [71]	-	⋆	⋆	⋆	⋆⋆	⋆	⋆	⋆	(8)
Choi et al. / 2019 / South Korea [7]	⋆	⋆	⋆	⋆	⋆⋆	⋆	⋆	⋆	(9)
Panhwar et al. / 2019 / USA [2]	⋆	⋆	⋆	-	⋆⋆	⋆	N/A	N/A	(6)
Card et al. / 2020 / UK [72]	⋆	⋆	⋆	⋆	⋆⋆	⋆	⋆	⋆	(9)
Gauravpal S. Gill et al. / 2020 / US [75]	⋆	⋆	⋆	⋆	⋆⋆	⋆	⋆	⋆	(9)

BMI: Body mass index; ACS: Acute coronary syndrome; IBD: Inflammatory bowel disease; HTN: Hypertension

Table S2. The Newcastle-Ottawa Scale (NOS) for assessing the quality of cross-sectional studies

**Study**	**Selection**				**Comparability**	**Outcome**		**Score**
	**Sample representativeness**	**Sample size**	**Non-Respondents**	**Ascertainment of the exposure (risk factor)**	**Comparability**	**Assessment of the outcome**	**Statistical test**	
Archimandritis et al. / 2002/ Greece [60]*	⋆	-	*	**	*	**	-	(7)
Kuy et al. / 2014 / USA [67]	⋆	⋆	⋆	⋆⋆	-	⋆	-	(6)
Barnes et al. / 2016 / USA [69]	⋆	⋆	⋆	⋆⋆	⋆⋆	⋆	⋆	(9)
Mendelsohn et al. / 1995 USA [59]*	⋆	-	⋆	⋆	*	⋆⋆	⋆	(7)

Table S3. The Newcastle-Ottawa Scale (NOS) for assessing the quality of case-control studies

**Study**	**Selection**				**Comparability**	**Exposure**			**Score**
	**Adequate definition**	**Representativeness of the cases**	**Selection of Controls**	**Definition of controls**	**Comparativeness of the cases and controls on the basis of the design or analysis**	**Ascertainment of exposure**	**Same method of ascertainment for cases and controls**	**Non-Response rate**	
Zakroysky et al. / 2015 / USA [68]	⋆	⋆	⋆	-	⋆⋆	⋆	⋆	⋆	(8)
Ehrenpreis et al. / 2016 / USA [70]	⋆	⋆	⋆	⋆	⋆⋆	⋆	⋆	⋆	(9)
Le Gall et al. / 2018 / France [22]	⋆	⋆	⋆	⋆	⋆	⋆	⋆	⋆	(8)
